# Social Workers’ Reports on Needs and Recommendations to Enhance School Safety

**DOI:** 10.3390/bs15050627

**Published:** 2025-05-04

**Authors:** Natalie Fensterstock, Kate R. Watson, Luz E. Robinson, Vanessa R. Warri, Anthony A. Garcia, Chaoyue Wu, Sawyer Hogenkamp, Yinuo Xu, Hannah Garner, Danielle Dunn, Ron Avi Astor, Dorothy L. Espelage, Susan D. McMahon, Linda A. Reddy, Andrew Martinez, Eric M. Anderman, Frank C. Worrell

**Affiliations:** 1 Department of Social Welfare, University of California, Los Angeles, CA 90095, USA; krwats@g.ucla.edu (K.R.W.); vwarri415@g.ucla.edu (V.R.W.); cywu1018@g.ucla.edu (C.W.); sawyerhogenkamp@ucla.edu (S.H.); daniellemdunn@g.ucla.edu (D.D.); rastoravi@ucla.edu (R.A.A.); 2 School of Education, University of North Carolina at Chapel Hill, Chapel Hill, NC 27599, USA; luzeli@unc.edu (L.E.R.); yinuo.xu@unc.edu (Y.X.); hannah.garner@duke.edu (H.G.); espelage@unc.edu (D.L.E.); 3 Graduate School of Education, University of Pennsylvania, Philadelphia, PA 19104, USA; gargar@upenn.edu; 4 School of Education and Information Studies, University of California, Los Angeles, CA 90095, USA; 5 Department of Psychology, DePaul University, Chicago, IL 60614, USA; smcmahon@depaul.edu; 6 Graduate School of Applied and Professional Psychology, Rutgers University, New Brunswick, NJ 08901, USA; lreddy@gsapp.rutgers.edu; 7 Center for Court Innovation, New York, NY 10018, USA; anmartinez@nycourts.gov; 8 Department of Educational Studies, The Ohio State University, Columbus, OH 43210, USA; anderman.1@osu.edu; 9 Berkeley School of Education, University of California, Berkeley, CA 94720, USA; frankc@berkeley.edu

**Keywords:** school safety, school social work, student mental health, qualitative

## Abstract

Issues with school safety and violence have raised concerns that warrant attention from researchers, policy makers, and practitioners alike. In this study, we explored how school social workers—a group of school personnel who are understudied in the school violence literature—experience school safety and violence in the United States. Using a sample of 271 school social workers, we analyzed qualitative responses to two open-ended survey questions: (a) What are the most concerning safety issues facing educators and staff in your school? (b) What policies, procedures, resources, or interventions are needed to better prevent or address violence in your school? Data were collected online between March and June 2022 by the American Psychological Association Task Force on Violence against Educators and School Personnel. Derived using a socioecological framework, findings reflect school social workers’ needs for workplace safety and perspectives on how to prevent and mitigate school violence at the school site, district, and community levels. Implications range from promoting strong relationships in schools and between the school and community to bolstering funding sources that can sustain programs, training, and staffing aimed at improving experiences for all students. The implications of our findings for future research and practice are presented.

## 1. Introduction

### 1.1. Social Workers’ Reports on Needs and Recommendations to Enhance School Safety

Student well-being and behavioral health are growing areas of concern in the wake of the COVID-19 pandemic (E. A. Jones et al., 2021; McMahon et al., 2024b; Watson et al., 2022). School social workers play a vital role in promoting student well-being and addressing behavioral health challenges in educational settings. Given their unique position, understanding school social workers’ perspectives on school safety and effective interventions for school violence is crucial to supporting student well-being. We explored the qualitative responses of school social workers in the United States on (a) malleable contextual factors that contribute to school violence and (b) responses or interventions that could mitigate violent incidents in schools. Insights from these frontline professionals can inform school safety practices and policies.

### 1.2. Safe and Supportive Schools as a Pressing Issue for Staff, Students, and Community

School safety is a critical issue in the United States and internationally (Astor & Benbenishty, 2019; Cohen, 2021; McMahon et al., 2022a, 2022b) because safety is closely associated with students’ ability to learn in schools (Benbenishty et al., 2016; Berkowitz et al., 2017). Recent data also underscore the significance of enhancing safety in these settings. According to the National Center for Education Statistics (2021), during the 2019–2020 academic year, approximately 77% of public schools reported one or more incidents of school violence, totaling an estimated 1.4 million incidents.

Widespread victimization in schools may substantially undermine the well-being of students, the school staff, and the broader community. Exposure to unsafe school environments is associated with increased mental health issues among students, negatively affecting their focus, engagement, and overall academic outcomes (Benbenishty et al., 2016; Chen et al., 2021; Côté-Lussier & Fitzpatrick, 2016; Duru & Balkis, 2018). In a recent national study conducted after COVID-19 in 2022, rates of violence and aggression against school social workers, psychologists, and school counselors were high, with 63% reporting at least one incident of verbal and threatening aggression from students, parents, or caregivers and 50% reporting at least one incident of physical violence from students (McMahon et al., 2024b). Indeed, feeling unsafe in schools has serious implications for school staff members, because it is linked to reduced job satisfaction, high turnover rates, and ultimately, leaving the profession (McMahon et al., 2024a, 2024b; Peist et al., 2020). Furthermore, schools serve as community hubs, and disruptions in school safety can weaken school and community cohesion and contribute to increased incidents of violence across settings (Cohen et al., 2009). Therefore, enhanced understanding of and targeted interventions in school climate and safety are essential—not only for direct stakeholders but also for the broader community (Bradshaw et al., 2021; Kutsyuruba et al., 2015). However, despite school social workers’ central role in mental health promotion and violence prevention, studies have rarely integrated their perspectives on school safety (e.g., Capp et al., 2020a, 2020b).

### 1.3. Role of School Social Workers in Enhancing School Safety

Most school social workers report working with a diverse caseload of students who exhibit a range of mental health, academic, and behavioral issues (Allen-Meares, 1994; Kelly et al., 2010, 2015). School social workers often work directly with students exhibiting emotional or behavioral challenges, making their perspectives on school- and student-related safety and well-being critical to forging a comprehensive understanding of school safety. Indeed, in a meta-analysis, Turanovic and colleagues (Turanovic et al., 2020) found that antisocial behavior, attitudes, and peer rejection among students were among the strongest predictors of school violence, including aggressive behavior and bullying. However, research on school social workers’ perceptions of school-level violence reduction policies is limited. Although social workers participate in the implementation of school safety programs (Astor et al., 2005; Pitner et al., 2017), there is a dearth of contemporary research exploring their perspectives on safety issues and responses (Cuellar et al., 2017).

The pandemic and broader sociopolitical climate surrounding 2021–2022 exacerbated mental health challenges and increased reported stress for students, families, and school staff members (E. A. Jones et al., 2021; McMahon et al., 2024b; Watson et al., 2022). Reports of school violence significantly worsened once schools reopened and resumed in-person sessions (McMahon et al., 2024a, 2024b). Immediately following the pandemic, infusions of federal funding (e.g., American Rescue Plan Act of 2021, 2021) allowed schools to hire social workers and expand mental health services (Griffith, 2021; C. Jones, 2021). However, the funding was primarily time-limited, and much of it has since been depleted (Fensterwald, 2021; Márquez Rosales, 2024). Many questions remain about whether funding streams will be renewed or sustained. The COVID-19 crisis underscored the critical role of school social workers in providing comprehensive schoolwide mental health support.

### 1.4. A Socioecological, School-Centric Approach to School Safety

Astor and Benbenishty’s (2019) ecological model for the study of school violence, safety, and climate was used to guide this analysis. This model positions the school at the center of a complex system composed of various interconnected contexts, including the school’s immediate environment (local community) and stakeholders (e.g., students, educators, and families), broader societal factors (sociopolitical trends, events, and policies), and place in time (historical and current events, time of year, etc.). The interplay of these internal and external forces shapes the school environment, influencing factors of safety and student and staff well-being.

Research on school safety tends to focus on student experiences of violence and outcomes, with perspectives of school staff members mainly used to triangulate student experiences. Research on educator perceptions of school violence is lacking, even more so for school personnel members who are not teachers, such as school social workers (Astor et al., 2010; Berkowitz et al., 2017; Capp et al., 2020a, 2020b). There is value in centering school social workers’ perceptions and experiences because they, too, are affected by school violence and are primary agents of school safety with responsibility to ensure the safety and well-being of students. In addition, school social workers can feel unsafe in schools, affecting their physical and mental health, job performance, and likelihood of staying in the field (McMahon et al., 2022b).

### 1.5. The Current Study

In the current study, we examined the experiences of school social workers in the United States, their descriptions of the scope of school violence, and their recommendations for increasing school safety. To this end, we employed a rigorous qualitative analysis of school social workers’ experiences and examined the following research question: What do school social workers identify as the most concerning safety issues facing educators and staff members in their schools and what do they believe is needed to better prevent or address such issues?

## 2. Method

### 2.1. Participants

The sample for this study featured 271 school social workers who answered at least one of two open-ended questions: (a) What are the most concerning safety issues facing educators and staff in your school? (b) What policies, procedures, resources, or interventions are needed to better prevent or address violence in your school? Most respondents (88.93%) identified as women; 9.59% identified as men and 1.48% identified as nonbinary or another gender (see Table 1 for demographic information). Most respondents identified as White (78.23%), followed by African American (10.70%), multiracial (5.53%), Hispanic (4.80%), and Asian (0.74%). Social workers had a wide range of school social work experience: 1 year or less (8.86%), 2–5 years (25.09%), 6–10 years (16.97%), 11–15 years (15.50%), 16–20 years (11.07%), 21–25 years (7.75%), 26–30 years (4.80%), and 31 or more years (2.95%). An additional 7.01% did not report their years of experience. Their grades and school levels are reported in Table 1. Social workers reported serving a wide range of communities, with 39.86% in suburban areas, 39.11% in urban areas, and 21.03% in rural areas. The sample was geographically distributed across the United States, with 40 states represented.

### 2.2. Data Collection

Data for this study were collected between March and June 2022 via a national survey by the American Psychological Association Task Force on Violence against Educators and School Personnel (McMahon et al., 2022a, 2022b, 2024a, 2024b). Through collaborations with professional associations representing teachers, school psychologists, school social workers, and other school personnel, a link to an online survey was sent to prospective participants. MCH Strategic Data also provided a subset of school staff emails, which were stratified by role (i.e., teacher, administrator, other staff member), U.S. region (i.e., West, Midwest, South, Northeast), urbanicity (i.e., urban, suburban, and rural), and school level (i.e., elementary, middle, high school, and combined). The complete sample included participants from all 50 states and Puerto Rico. The qualitative responses from school social workers informed the development of our research question and were analyzed for the current study.

### 2.3. Analytic Approach

The coding process used thematic analysis, which allows for flexibility in the recognition and development of themes (Braun & Clarke, 2006). This method enables analysts to both uncover and interpret patterns using a combination of inductive and deductive strategies. Analysis began with inductive coding of responses from a random 10% sample of participants, followed by development of a coding frame and manual that included broad categories, codes, and subcodes. The codebook was then tested on a second random 10% sample of participant responses, after which it was finalized for use on the remaining data. Coder training was provided, including the use of a deductive approach, rationale for when double coding was acceptable, the method for splitting responses that illustrated different codes, and examples of each code (Braun & Clarke, 2006). An example from the codebook is shown in Table 2.

Coding was performed by a team of four researchers who worked in pairs. Across both pairs, each person independently reviewed half of the data, then convened to compare their coding results with a partner who coded the same half of the data. Together, the two pairs coded the full data set and intercoder reliability is based on the full data set. Intercoder reliability was determined manually using percentage agreement (O’Connor & Joffe, 2020). The two pairs achieved 90–93% agreement. Differences were resolved using a consensus approach. After consensus was reached, all quotes were transferred from individual coding templates into a final coding document. Each author had the opportunity to review the coded data and identify patterns of response that were then discussed among team members.

#### 2.3.1. Validity of the Qualitative Data Findings

The team reflected on connections between codes in and between the two questions studied. Each team member iteratively wrote analytic memos tracking their coding processes and verbally shared their key takeaways and reflections during coding meetings (Creswell & Poth, 2018; Saldaña, 2013). The team synthesized feedback from each team member to establish four themes. Throughout the analytic process, the full research team—including members of the APA Task Force on Violence against Educators and School Personnel—discussed the development and use of the codebook, development of themes, and presentation of valid findings. We also compared our findings to quantitative survey results as a form of data triangulation (e.g., McMahon et al., 2022b, 2024b). Our findings were similar to previously reported quantitative findings in that respondents reported significant concerns about parent and student aggression, staff burnout, and a need for more consistent discipline policies and additional training (see McMahon et al., 2024b).

#### 2.3.2. Researcher Reflexivity

This study was managed by academic researchers in social work and education programs in urban metropolitan areas. Our team has potential biases toward softening and asset-based responses (e.g., restorative practices) to school violence and victimization. Although we tried to remain neutral and give space to responses across the softening–hardening and rural–urban spectrums, we recognize that our biases may have informed how we decided to present findings and implications. The first author is a former K-12 educator who studies community schooling and systems of collaboration among school staff members, which informed their perspective on participant responses. Other authors have direct social work practice experience with youth in school settings, adding dimensions to our collective interpretations of the data.

## 3. Results

We identified four overarching themes related to the perceptions of school social workers regarding school violence and safety: (a) concerns about school safety require strengthening of school–community connections; (b) challenging student behavior requires interventions focused on building a strong school climate and community; (c) concerns about student discipline, disruption, and defiance can be addressed with consistency, accountability, and improved systems of communication; and (d) concerns about challenging student behaviors can be addressed with increased staffing and training. We structured our findings in accordance with the connection between the two survey questions examining the top safety concerns and recommendations of school social workers. Each theme spans responses to both survey questions and features subthemes regarding concerns and recommendations to address these concerns (see Table 3). This choice aligned with the socioecological approach to school safety (Astor & Benbenishty, 2019).

### 3.1. Theme 1: Concerns About School Safety Require Strengthening of School–Community Connections

School social workers reported that school safety concerns required strengthening of the connections between schools and their surrounding communities. Theme 1 encompasses subthemes that threats are primarily external to the school and the need for positive school–community connections.

#### 3.1.1. Concern: Safety Issues Seen as External to the School

School social workers viewed safety concerns as primarily external. Some concerns stemmed from parents and families or inadequate building security that enabled external issues to permeate the school. Participants frequently described experiencing “parental hostility”, “direct physical and verbal aggression from parents”, and “[parental] outbursts due to misunderstanding or lack of communication”. For example, one respondent remarked that “the parents … are increasingly becoming more adversarial to staff”. Participants also discussed parent mental health and substance use challenges as a heightened safety concern. One participant noted that parents sometimes posed safety risks to students by “coming into school to threaten or fight another student who had conflict with their child” or because they “brought weapons to campus”. These findings suggest that erosion in accountability and respect may lead parents and community members to act with impunity—generating potentially dangerous and negative consequences for both students and school personnel.

Respondents further reflected on the difficulties that arise from parental disengagement, including misalignments in perceptions of who is responsible for student behavior and relationship development between school personnel and parents. One participant shared that parents are “blaming the schools for all social problems and expecting school staff to fix students”. Another respondent acknowledged that the demanding work schedules of parents “to make ends meet … [affects] their ability to know their child and address their needs effectively” and may not allow “reinforcing school expectations in the home due to competing priorities”. Participants said that parents could do more to intervene in students’ aggression toward other students and school personnel.

In addition to external safety risks imposed by parents, school social workers also spoke about safety concerns associated with the broader community and neighborhood violence. Respondents mentioned the presence of “open gang activity”, “community violence spilling over into the school”, and a “lack of monitoring gang and drug activity” in school. Respondents also connected poor campus and building security with heightened safety concerns about parents, community members, and outsiders having unfettered access to the school.

#### 3.1.2. Recommendation 1: Need for Stronger School–Community Connections to Enhance Safety

Participants identified a need for greater collaboration between schools and communities aimed at filling gaps in support for students’ behavioral and psychosocial needs. One respondent suggested “more community resources for family and community members to address factors related to drug use, gang involvement, housing instability, and unemployment”, whereas another noted that “stronger community and parental engagement” and “working with local organizations, mentoring groups, and public service agencies to connect families with resources and support and to address gang violence and drug use” would benefit the school. These findings highlight a need to work in the community to identify and cultivate relationships between community organizations and schools to ensure students and their families receive needed support.

#### 3.1.3. Recommendation 2: Need for More Positive School–Family Connections

One participant proposed the following recommendation to enhance school–family connections before children enter kindergarten: “More early childhood interventions within the community setting so relationships between school and families are already existing.” Another respondent suggested, “Somehow parents need to be addressed about how they interact with school staff. … We truly love our students and try to support in the best way we can, yet we are constantly seen as the ‘bad guy.’” These examples highlight the critical and ever-expanding role of schools in the lives of children and their families, suggesting that more work is needed to create a paradigm shift in how parents and families conceptualize the roles of schools and their personnel in support of students.

### 3.2. Theme 2: Challenging Student Behavior Requires Interventions Focused on Building Strong School Community and Climate

Respondents reported that addressing difficult student behaviors, most notably physical and verbal aggression, would require interventions focused on building a stronger school climate and community. Theme 2 includes subthemes related to students presenting with complex behavioral challenges and an emphasis on verbal and physical aggression, along with recommendations that schools honor how complex behavioral challenges require multitiered ecological approaches and stronger student–staff and student–student relationships.

#### 3.2.1. Concern: Students Present with Complex Behavioral Challenges

School social workers identified student verbal and physical aggression as significant challenges for school personnel. When describing the challenges of students’ verbal and physical aggression, many participants highlighted gaps in self-regulatory skills for students who present with complex behavioral challenges. Participants generally described verbally and physically aggressive students as “emotionally dysregulated”, lashing out at other students and school personnel due to “emotional outbursts”, and “hostile students who suffer from trauma and other mental health struggles that are not receiving appropriate support services”. Participants discussed the verbal aggression of students in terms of “yelling and swearing at the teacher”, “using threatening language”, “students texting each other to go fight in the bathrooms”, and cyber-bullying—including online threats to bring weapons to school. Respondents reflected on the impact of such forms of verbal aggression from students on school personnel. One participant commented, “We have had a lot of students make threats this year such as, ‘I am going to burn the building down.’ … This worries staff, and they feel as though sometimes it is not taken seriously.” These findings suggest that student verbal aggression may have as significant of a negative impact on the mental health of school personnel as physical aggression.

In addition to experiences with verbal aggression between students and between students and staff members, respondents noted more extreme safety challenges with physical aggression. Participants described students as being “aggressive with objects” and “throwing books and other sharp objects”. One respondent shared a particularly concerning anecdote: “There has been an escalation of students with aggression and acting out. Staff has fears since in our district a teacher was strangle[d], raped, and found unconscious.” Another participant spoke of “injuries resulting from handling violent or out-of-control students”. These findings illustrate the critical need for more holistic approaches that support students’ behavioral and emotional development to address safety concerns related to students who may engage in increasingly dangerous behaviors toward other students and school personnel.

#### 3.2.2. Recommendation 1: Complex Behavioral Challenges Require Ecological Approaches

Respondents reflected on the dual challenge of addressing dysregulated and aggressive students with limited staff and family support and dealing with the limited resources available to them from their schools. In addition to noting these challenges, they emphasized the need to “meaningfully connect with anyone in the school or community” and for “more work on school-wide climate and staff well-being”. They suggested increased “support of changing the culture. Everyone complains but very few are willing to put in the work to actually address and start to fix the issues”. Another respondent explained, “We need more programs to engage kids in school and find a sense of belonging.” This subtheme demonstrates a critical need for both stronger multilevel approaches that attend to the development of the whole child and a larger staff presence to support these approaches.

#### 3.2.3. Recommendation 2: A Need to Develop Stronger School-Based Relationships

Given the reported difficulties in forming meaningful relationships at school, respondents emphasized the importance of providing teachers with “opportunities [and] time … to form positive relationships with their students (and less focus on time-wasting paperwork)”. One respondent stated that “when students feel the moral compass of doing what is right to protect others, they will be more prepared to keep the school and fellow students safe”. One participant suggested that better engagement with students could involve including “more student voice that represents the actual make-up of the student population”. Respondents also noted the importance of productive relationships between staff members. One participant, for example, called for “more opportunities for consultations between staff–teachers and leadership–professionals (i.e., counselors, seasoned educators)”. These findings suggest the need for more comprehensive approaches to school-based relationships across the board by increasing opportunities for interpersonal connections, supportive staff–student interactions, and promotion of socioemotional well-being among students.

### 3.3. Theme 3: Concerns About Student Discipline, Disruption, and Defiance Can Be Addressed with Consistency, Accountability, and Improved Systems of Communication

Participants indicated that concerns regarding student discipline, disruption, and defiance could best be addressed through consistent responses from the school that ensure students are held accountable for their actions. They identified improved communication by the school staff and administration as one mechanism to achieve the desired outcomes. Theme 3 includes subthemes related to individual and school system capacity: uneven distribution of consequences, lack of communication regarding incidents, lack of training in advanced disciplinary approaches, and a need for behavioral support and stronger schoolwide systems to navigate discipline.

#### 3.3.1. Concern 1: Uneven Distribution of Consequences

School social workers described how school staff members, including teachers and administrators, responded differently and often inconsistently to incidents involving student behavior. Some participants shared that there is “poor accountability for behavior” and a “lack of follow through with discipline”, whereas others noted that their school has “violence with *no* [emphasis in original] consequences”. One participant remarked that “students have been reported for putting stuff in their teachers’ food and drinks without any care of the consequences”. Respondents also noted the mismatch between behavior and consequence was due to insufficient staffing support and time to understand and address individual incidents adequately. Moreover, some respondents explained how student behaviors such as throwing objects at other students is seen as “joking and playing” and how “many times, these actions go unpunished at an administrative level”. In addition to reflecting on the lack of appropriate and consistent disciplinary response for student behavioral incidents, participants indicated that certain school policies regarding discipline fell short. For example, one participant explained:

When students become escalated (yelling and swearing at the teachers), teachers have been told they need to keep [the students] in the classroom or allow them back in, despite the safety risk to the students in the classroom and the teachers.

Another participant summarized, “Sometimes things are not taken as seriously as they should be and the consequence does not match the severity of the behavior.” Respondents emphasized a general lack of student accountability, discipline, and respect that fostered unsafe school environments for school personnel and inconsistency in how the school’s administrative team handles discipline.

#### 3.3.2. Concern 2: Lack of Communication and Leadership Regarding Incidents

Many participants remarked on a lack of communication or inconsistency regarding administrative responses to discipline issues. Broadly, participants noted how “inconsistent messages [are] given” and how there is “a lack of clear communication between administration and faculty regarding policies when crises occur”. For example, one respondent explained how “information trickles down from admin[istration] to staff and varies building by building”, whereas another respondent shared the following:

Staff are not informed when there is an issue or a threat. … We are also not clear in the expectation for discipline between administrators. One admin[istrator] will be more lenient with violations than others. [This] doesn’t set a good precedent.

Several respondents were more pointed with their responses, explaining that an overall “lack of effective leadership”, “lack of support from administrators”, and “horrible, ineffective, unhelpful, unkind, not understanding principals and school social work leadership” negatively affected school safety.

In addition, participants also described how lack of timeliness regarding requested support for de-escalation among students also contributes to overall school safety concerns. One school social worker indicated that teachers “call for support and it is not always available or support is late in responding”. Some respondents said that their schools did not conduct threat assessments in an effort to improve overall school safety. One participant perceived that inadequate administrative support drives poor mental health among school personnel members, creating “an environment that is not comfortable for all involved”. These findings suggest that inconsistent administrative support and ineffective de-escalation strategies may be not only perpetuating unsafe school environments but also contributing to poorer mental health outcomes.

#### 3.3.3. Recommendation 1: Need for Stronger and More Transparent Systems to Navigate Discipline

Participants expressed the need for administrators and fellow staff members to implement streamlined processes and systems to respond to student behavioral concerns. For example, one respondent expressed a need for more consistent disciplinary responses with “buy-in and follow through from all staff”. Another said, “Admin needs to document the violent incidents. The information is hidden from the district and it appears to be a ‘safe’ school but it is not.” A third respondent called for the following:

Consistent procedures from the district level administrators. The administrators who work at the district office need to visit schools and classrooms on a regular basis. Many of them have no idea about what is happening at the schools. These administrators are making decisions without knowledge.

Despite a clear call for consistency, respondents vacillated in the type of consequences. Some respondents requested the maintenance of zero-tolerance policies, “harsher punishments for those who engaged in physical altercations”, the ability to suspend elementary students for violent behavior, and generally stricter interventions. Others called for more adaptive policies for less egregious incidents such as providing alternative clothes for dress code violations instead of sending students home. Other participants echoed these sentiments, requesting consistency with procedures from the district level and accountability for administration regarding clarity in discipline policies.

#### 3.3.4. Recommendation 2: Need to Develop Stronger Staff Communication and Support

Although the level of desired strictness shifted across respondents, a distinct throughline signaled the need for clear and consistent communication among staff members at various levels of the school structure (teachers, support staff members, clinicians, and administrators). Respondents expressed the need for consistent support, mainly from school administrators, regarding accountability for disruptive, sometimes violent behaviors among students. Respondents also noted the need for greater collaboration among school staff members to better address student disciplinary challenges. Participants discussed the need to “provide more opportunities for consultations between staff [and] teachers and leadership [and] professionals (i.e., counselors, seasoned educators)”. Other respondents reflected similarly, noting that staff members should begin “working together to problem solve while holding students responsible” and “get everyone on the same page as to what disciplinary procedures are so there is cohesion in what everyone is doing”. One suggestion for developing stronger staff connections was “top-down respect, non-threatening management. Motivational supervision with focus on the positives”. Other respondents discussed the role of building trust and transparency between administrators and teaching staff members to develop a culture supportive of openly addressing student behavior and teachers’ concerns with incidents.

### 3.4. Theme 4: Concerns About Challenging Student Behaviors Can Be Addressed with Increased Staffing and Training

School social workers reported feeling ill-equipped to respond to challenging student behaviors and said this issue could be partially addressed with adjustments to staffing, training, and funding. Theme 4 includes subthemes related to the overwhelming nature of the multiple responsibilities of school social workers and a lack of training in advanced disciplinary approaches, leading to recommendations of hiring additional staff members, offering specific trainings, and bolstering special education staffing and support.

#### 3.4.1. Concern 1: Staff Members Feel Overburdened in Their Roles and Schools Are Understaffed

Respondents described struggling with mental health due to a heavy workload and concerns about job stability. They reflected on the “burnout”, “mental exhaustion”, “personal emotional [and] mental health drain”, and “stress” of working in schools today. Another participant shared that “staff are overwhelmed and overworked”. Although this feeling of being overwhelmed is problematic alone, participants also noted that feeling overburdened in their role “causes them to not have the time, energy, skills, and patience to best support the students”.

Participants linked concerns about staff mental health, burnout, and role overload to increased workloads and understaffing. They also connected understaffing with turnover and difficulty retaining staff members. Some respondents explained how administrative and staff turnover led “to the overall feelings of chaos” and “instability” in the schooling experience for students and staff members alike. “We are scrambling”, another shared. Respondents reflected how general “staff shortages make the environment unsafe. … There are not enough staff to supervise students safely”. Respondents also mentioned specific staffing shortages, including the need for more school resource officers and security guards stationed at one school for more days per week as opposed to spread thin across multiple schools: “We don’t have the resources or training to deal with students with violent behaviors.” Responses such as these linked the presence of school security personnel with the capacity to respond better to student behaviors. In a different vein, participants also lamented the lack of school social workers, therapists, direct care staff members, school nurses, and employees adequately trained in behavior management, socioemotional learning, restorative practices, prevention, and intervention. They broadly implied that schools are “scrambling” due to insufficient staffing.

#### 3.4.2. Concern 2: Lack of Training with Advanced Disciplinary Approaches

Beyond a struggle to communicate among staff members and consistently implement disciplinary responses to student behavior, participants also reflected on the lack of training in advanced disciplinary approaches. Some respondents noted a general “lack of support in managing behavior from the district level” and a recognition that they “don’t have the resources or training to deal with students with violent behaviors”. Other respondents spoke of inadequate responses to student behavior connected to a lack of understanding and training:

[Staff members] do not understand the long term effects of trauma. We were told about trauma and ACES [adverse childhood experiences], and that is considered being a Trauma Informed District… Students are often re-traumatized, blamed rather than listened to, and staff and administrators do not respond to concerns or recommendations for those affected by trauma.

Additional reflections included a lack of training to respond to mental health emergencies. Even when protocols were in place, implementation was sometimes ineffective, suggesting the need for additional support. One respondent explained how “the holds that were taught to us during instances in which there is an immediate threat of harm to self or others are not always effective”. Respondents also highlighted the need for support with “restorative justice, social emotional learning, and teacher coping skills”, alongside calls for supporting students and staff members with “effective communication, boundary-setting, and emotional recognition and regulation”. This finding suggests that the current provision of mental health interventions in schools may be inadequate for addressing student behavioral and developmental needs.

Respondents also noted challenges navigating inclusive general education settings and the “least restrictive environment” guaranteed for students with special needs through the free appropriate public education clause in the Individuals with Disabilities Education Act (Individuals with Disabilities Education Act of 1990, 1990). Respondents reflected on “physical and verbal aggression from special education students in the mainstream setting” and general “behavioral challenges in the least restrictive and safe environment”. One respondent reported challenges with “managing individuals with developmental [or] intellectual disabilities”, whereas another lamented having “no protection against special needs students who hit, punch, bite, pull hair, etc., to staff”. They continued by sharing how they “have a colleague who has permanent damage to the nerves in her hand from a student bite”.

#### 3.4.3. Recommendation 1: Hiring Additional Staff Members in Various Roles

As previously mentioned, respondents reflected on the need to hire additional staff members for various positions their schools. Respondents also noted the role of funding in hiring and providing more robust resources and training. Several responses indicated the need for increased funding to “hire more support staff” and focus the time of support staff members on one school site. For example, one respondent reported the following:

[School resource officers and school social workers] are vital to the safety of students and staff. There are multiple times I can think of over the years in which an incident has occurred when neither I nor [*sic*] the [school social worker] were in that school, because we were serving other schools and were not able to get there to perform our duties.

Respondents also noted the need to fund clinical resources and increase salaries to “entice staff to come to our schools”.

Just as respondents were clear about their sentiment regarding understaffing, they were also clear about the roles that needed to be added or staffed more robustly. Respondents requested that schools hire more support staff members, clinicians, counselors, school social workers, and behavioral specialists. Respondents also made general calls for “more staff”, better staff-to-student ratios, and smaller class sizes. They explained that staff members who were hired for these roles needed to be “trained properly” and able to ensure the “fidelity of interventions”. Participants also noted that mental and behavioral health staff members needed to be leveraged more effectively at their school sites, including to “build the capacity of others to respond to behaviors”. Such responses indicate the need to hire particular staff members to support the complex needs of students regarding behavior and mental health. Some respondents also called for the hiring of additional administrators such as vice principals and restorative deans (e.g., specialists in restorative justice and student behavior) to address student behavior. In addition to calls for more staffing related to behavioral and mental health support, respondents also noted the need for more school resource officers or school police. Several participants said hiring more campus security professionals would aid in making school environments safer. One participant demonstrated remorse in this regard, sharing how they “hate to say it, but … [I] would appreciate more police presence”. This response aligned with calls for “more police officers” and “better trained security officers”. Another participant noted the role of relationships and security, explaining how their school needs “a full time School Resource Officer … [to] have more time to proactively connect with students when [a] crisis isn’t happening”. Finally, participants reflected on the need for safety and crisis teams to respond to acute circumstances involving school safety. Such reflections bolster the call to increase the presence of adult staff members on school campuses but differ from prior responses regarding the type of staff presence requested. These examples also point to the need for comprehensive safety strategies that address overall challenges to the safety and well-being of students and school personnel.

#### 3.4.4. Recommendation 2: The Need for Specific Trainings to Support Staff Effectiveness

In addition to requests to hire more staff members across roles and skillsets, respondents also noted the need to train current staff members in how to handle specific student behaviors and experiences. They reflected on the usefulness of training to respond to childhood trauma and the need to strengthen de-escalation tactics and general skills related to navigating the complex interpersonal relationships common in school settings.

Respondents requested training in three broad areas—training in general, mental health training, and behavioral health training—and five precise areas—socioemotional learning, tiered interventions such as a multitiered system of support, restorative practices, trauma-informed practices, and threat assessment and crisis prevention, including de-escalation tactics. Many respondents requested training on multiple related topics. For example, they tied requests for restorative justice information to requests for training in positive behavioral interventions and supports, a tiered approach used to promote a healthy school culture and climate. Similarly, requests for training in socioemotional learning were tied to broader requests to emphasize mental health in schools through ongoing staff training. These interconnected requests indicate the potential power of layered approaches of support for staff members to holistically support the student experience and address complex behavioral needs. Moreover, it is important to note the connection among a perceived lack of training, requests for training at school sites, and requests for staff members with specific training in response to meeting the behavioral struggles of students, as evidenced by such comments as “there needs to be more training opportunities, space for training, and more social workers and support staff” and the need for “more staff available for restorative interventions so teachers can continue teaching while behaviors arise”. These social workers identified gaps in their capacity as individuals and their broader team’s capacity in collaboration with other school staff members, and they asked for support to do their job more effectively.

#### 3.4.5. Recommendation 3: Bolstering Special Education Staffing and Support

Social workers’ responses addressed students with special needs, including the need for resources and training to support such students adequately. This recommendation reflects the perceptions, experiences, and responses shared by staff members who worked with students with special needs. Responses spanned topics of engaging with the least restrictive classroom environment, special education funding, and concerns from staff members at schools dedicated to serving students with special needs.

Challenges reported regarding students with special needs coincided with requests to implement trauma-informed care approaches and provide “better support and increased staffing for mainstreaming special education students”. Participants also called for hiring behavioral interventionists focused on supporting behavior plan implementation for students with special needs and increasing staffing of special education paraprofessionals who provide one-on-one support in and beyond the general education classroom.

Additionally, respondents reflected on the role of funding in supporting students with special needs. Participants explained how special education is not fully funded, resulting in students not receiving the support they need. One participant suggested providing “extra pay for special education staff who have to engage with potentially aggressive students”. Last, a few respondents described their experiences working at schools with a focus on students with special needs. One participant shared how “the largest safety issue for school staff is physical violence from students who are emotionally dysregulated”. These respondents said understaffing in their schools exacerbated their struggles with student behavior. One participant explained that “because of the lack of staff, staff are over reactive [*sic*] instead of having the time to focus on proactive strategies”.

## 4. Discussion

As our findings show, school social workers reported threats to school safety and proposed recommendations across multiple ecological levels. Social workers were concerned with school–community relations, interpersonal interactions in the school, and school and district measures to enhance school safety and well-being for staff members, students, and their families. Themes from this study align with Astor and Benbenishty’s (2019) ecological model of school violence, safety, and climate. The model supports the social work perspective of assessing a person in their environment, emphasizing the interconnectedness of individuals and their surroundings (Teater, 2014). The school, as the central focus of Astor and Benbenishty’s (2019) model, is situated in a complex system of interrelated factors. These factors include the immediate school environment, broader community, and sociopolitical context of the current time period. The model highlights how external influences, such as family dynamics, neighborhood characteristics, and societal trends, interact with factors in the school, including school policies, leadership, and classroom climate. The reciprocal relationship between the school and its environment underscores the importance of considering multiple levels of influence when addressing safety-related issues.

Social workers expressed concern about the mental health of both students and staff members following COVID-19. Youth mental health significantly deteriorated during the pandemic. During this period, many young people experienced social isolation, disrupted routines, and increased reliance on digital platforms for connection (E. A. Jones et al., 2021). This crisis led several U.S. medical associations, such as the American Academy of Pediatrics (2021), to declare a state of emergency in child and adolescent mental health. The pandemic also negatively affected the mental health of adults, including parents and school staff members (Hatzichristou et al., 2021; Murata et al., 2021). Our respondents reported increased stress, overload, and difficulty meeting the increased challenges of their jobs upon returning to school, with parents, students and teachers still dealing with the traumas they experienced during COVID-19. Although this study focused on data from the United States, we believe the findings are applicable to conversations about school safety, staff collaboration, and mental health across the world.

### 4.1. Schools Need to Strengthen Connections with the Broader Community

Respondents repeatedly emphasized the importance of developing strong connections between the school and the broader community. They described these connections as a mechanism to develop trust and rapport among the adults in a student’s life and ultimately, to develop a sense of safety across the multiple environments a student experiences. School social workers can support this goal through assessing the needs of their students and families, identifying local community organizations that can best serve those needs, and establishing relationships with these organizations (Tan & School Social Work Association of America, 2024). Such relationships can bridge the gap in service access for students and their families, thus demonstrating the role of schools in directly supporting the whole family (Epstein & Salinas, 2004; Haig, 2014; Schwartz et al., 2023). By building relationships with community organizations and leveraging these connections to support families, school social workers can weave an intricate web of trust and rapport among students, families, schools, and community organizations; they can support families in building their own system of care (Early Childhood Learning and Knowledge Center, 2013; Maier et al., 2017). Moreover, these connections and rapport can serve as an entry point for families to become involved in the school community, developing a shared understanding of the goals for their student’s success with a broad coalition of school staff members (Chavkin, 2017). Future research could explore the connections among establishing community partnerships, students’ and families’ experiences with their school community, and students’ related academic and socioemotional outcomes.

### 4.2. Students’ Behavioral Challenges Require System Approaches

Participants in our study reported that students’ behavioral challenges required responses across the ecological spectrum, ranging from interpersonal interventions to broader, organizational adaptations. Empirical study of whole-school safety interventions or organizational factors related to school safety has been quite limited. However, prior schoolwide interventions that sought to address school climate and organizational capacity to sustain safety and prevention efforts have proven successful (e.g., Astor et al., 2021). Schools typically view evidence-based practices as the gold standard for intervention. However, there are numerous challenges with sustaining such practices long-term, including their cost, difficulties scaling interpersonal or small-group interventions to a whole school, inadequate testing of these approaches across diverse populations and contexts, and fragmented programming that results from implementing various programs without sufficient attention to programmatic integration and coordination (Adelman & Taylor, 2018; Fixsen et al., 2013; Nadeem & Ringle, 2016; Osher et al., 2021; Pinkelman et al., 2015).

Social work has consistently valued the implementation of ground-up responses in addition to evidence-based practices to ensure the contextual appropriateness of school-based programs (Astor et al., 2021; National Association of Social Workers, n.d.). As a result, we expected to see references to uplifting community voices or empowering students and staff members to identify and propose solutions among survey responses. However, social workers in our sample primarily focused on structural changes in schools and providing supportive services to children with behavioral and mental health issues. Current practice models for school social workers suggest a need to work across the ecological spectrum with families, schools, and communities to ensure the well-being of the school and its students. They can engage in universal prevention and intervention efforts and work with individual students to bolster academics, socioemotional wellness, mental health, and school climate (Tan & School Social Work Association of America, 2024). Although empirical research on school climate efforts has typically focused on the needs and perceptions of students, it is equally important for school staff members to experience the positive climate that they are charged with implementing (Capp et al., 2020a, 2020b). Researchers should continue to study school climate and community from diverse perspectives and seek to understand change mechanisms and causal directions between positive school climate and improved academics, belonging, and socioemotional wellness.

### 4.3. School-Based Relationships Are Important and Should Be Prioritized

Respondents emphasized the importance of school-based relationships, particularly between school staff members and students and among staff members. This recommendation is seen primarily in the second theme connecting school safety with the need to develop stronger school-based relationships. To date, much school-based relational research has concentrated on peer relationships, with a secondary focus on teacher–student relationships and negligible attention given to other school-based relationships (Kim, 2021; Littlecott et al., 2018). Peer relationships are believed to significantly affect young people’s psychological well-being, both positively and negatively (Balluerka et al., 2016; Gowing, 2019). In schools, peer relationships also contribute to the overall school climate (Traylor et al., 2016). Positive teacher–student relationships have been linked to improved student engagement (e.g., attendance, behavior, and achievement) and decreased problematic behaviors, suspensions, and dropout (Quin, 2017; Roorda et al., 2011). Both students and teachers have reported that supportive teacher–student relationships contribute to student well-being (Graham et al., 2016). Future research should broaden the conceptualization of school-based relationships to include relationships between school staff members and administrators, among staff members, and between nonteaching staff members and students.

### 4.4. Improved Systems of Communication Are Needed for Consistency and Accountability in Disciplinary Responses

Participants reflected on the difficulties related to striking a careful balance between promoting positive and safe classroom environments for all. They noted the tension between policies that encourage teachers to address student behavior through relationships in the classroom and policies that enable responses to student behavior outside of the classroom space (i.e., in the office, with an administrator). Such tensions elucidate the broad spectrum of approaches to proactively building safe and supportive classroom and school environments and responding to student behavioral incidents as they arise. Topics of classroom management and culture building in alignment with promoting safe school environments and navigating discipline are complex and interconnected, requiring comprehensive response structures.

Participants highlighted the importance of consistent discipline policy implementation, which relies on routine, clear communication among staff members and aligned responses to incidents (Irby & Clough, 2015; Winkler et al., 2017). A strong orientation toward relational communication from district and school leaders can support communication among staff members (Dupper, 2010; Kowalski, 2005), promote clarity with procedures and policies related to discipline and support with student behavior (Lewis & Sugai, 1999), and build effective communication patterns between families and schools (Epstein, 2010; Epstein & Salinas, 2004). Collaborative structures such as coordination of services teams, student support teams, and individualized education plan teams are examples of approaches designed to support individual student success by leveraging the expertise and collaboration of staff members across roles at a school site (Clark, 2000; Lai et al., 2016; Rosenfield et al., 2018). Such teams could include general education teachers, special education teachers, school administrators, mental health counselors, and other clinicians or experts. Although these collaborative structures can enable students to receive the services and supports both they and their families need, they are often focused on individual case management.

Future research could examine school site and district practices that leverage such multidisciplinary teams to support broader school safety and climate initiatives and move beyond individual student case management. Such studies should include an exploration of the role of communication among school staff members in and beyond these team structures to support the student experience. Further, whereas current individual student support teams are often focused on problem-solving, broader schoolwide teams could leverage an assets-based approach to improving school connectedness for all students.

### 4.5. Schools and Districts Should Collaborate to Address Staffing, Training, and Funding to Improve Safety

In both general education settings and schools focused on students with special needs, respondents noted understaffing, role overload, and a need for additional staff members to support students and promote a safer school environment for all. Further, to educate students with special needs in the least restrictive environment, schools need to hire specialized staff members and provide them with adequate resources and support to educate these students safely. Our findings point to the need to both improve the staff-to-student ratio and hire more staff members for specific roles related to student support and well-being.

Increased staffing typically requires additional funding or greater local control over funding streams, which often flows through the school district. Although school funding differs enormously across the United States (Morgan, 2022), it often consists of funding streams designated for specific purposes and that limit local control and decision making. Conversion to universal funding streams would allow local districts more control over how they prioritize funding (Tarnowski, 2024). Universal funding would be an important change because schools in different areas have different student populations and needs and should be able to target funding appropriately. Schools should be able to determine what staff roles are most needed locally and how to use their staff most efficiently.

As an alternative to hiring additional staff members, schools might be able to use their staff more efficiently. More efficient use of staff members would require both better training and a commitment to collaboration across roles (Adelman & Taylor, 2018). School staff members who work directly with students (e.g., social workers and teachers) are typically not leveraged as systemic thinkers, and they need to be trained as such to contribute to the holistic provision of support to the school, students, and families beyond interpersonal interactions. Such a change would also require a willingness on the part of school administrators to think outside of the box and allow social workers to expand their role beyond clinical interventions to universal interventions. This expansion of the social work role is in line with current best practices (Tan & School Social Work Association of America, 2024).

In addition to the need for additional staff members, there is also a need to recognize that schools typically experience tremendous turnover in staff and administrative positions and have difficulty hiring qualified candidates across all roles (Rodgers & Skelton, 2014). Turnover and hiring challenges were only exacerbated during the pandemic (Gecker, 2021; Heyward, 2021; McMahon et al., 2022a, 2022b, 2024b). Such challenges make adequate staffing difficult to maintain. Thus, school systems must dedicate additional time and resources toward retention and development of staff members. One low-cost approach to retention is mentoring. Mentoring of junior staff members by experienced staff members has been shown to increase staff retention and diversity (Gabbadon, 2023; Morettini, 2016). Team teaching and shared preparation periods can also support the retention of junior staff members (Rodgers & Skelton, 2014). Other strategies for retention include increasing financial support for professional training programs; streamlining licensing processes for teaching and other school professionals; raising substitute pay to attract qualified candidates; and expanding professional development opportunities (Carver-Thomas et al., 2021). Teacher training programs tend to underemphasize classroom management skills and strengths-based approaches to working with students (Oliver et al., 2011; Shank & Santiague, 2022). As a result, most new teachers struggle in their first years to manage their classroom and engage students effectively. Better professional development is needed both in teacher training programs and after educators have entered the field. This recommendation can be extended to all school-based professionals who are generally trained as experts in their fields but not in effective cross-role collaboration.

Because adequate funding from state, local, and federal governments is likely to remain an issue for schools, there is also a need to increase schools’ capacity to identify and maintain additional funding sources (Astor et al., 2021). The American Rescue Plan Act of 2021 (2021) provided a historic investment in U.S. schools and allowed for the expansion of school-based mental health services (Griffith, 2021; C. Jones, 2021). Funding was primarily time-limited and has since been depleted (Fensterwald, 2021; Márquez Rosales, 2024). Questions remain about whether these funding streams will be renewed or sustained. Schools must be creative about building capacity to ensure funding, training, and staffing levels that enable long-term viability. As one example, school staff members may be supplemented with graduate-level interns and connections to university service-learning programs. Organizational changes may also be needed to identify and secure additional funding. Many times, grants and other forms of funding are available, but schools may not be aware of them or have the resources to apply. Prior demonstration programs (e.g., Astor et al., 2021) have illustrated how schools can collaborate with universities to expand their capacity to develop and sustain increased staffing, training, and funding over the long term.

Future research should continue to assess how we fund and staff schools to address complex student needs and how schools can develop more holistic programming in an environment of one-off, time-limited funding. School climate and organizational research should also incorporate organizational and behavioral theory more comprehensively. Organizational behavior research has found that psychological safety and other aspects of positive organizational climate are important factors in job satisfaction and productivity (e.g., Edmondson, 2018). This awareness should be applied to schools.

### 4.6. Study Strengths and Limitations

This study should be understood in the context of its strengths and weaknesses. As a qualitative study, its findings were not meant to be generalizable. However, the broader study did collect information from a large convenience sample that included respondents from all 50 U.S. states. Due to the large sample size, findings may be transferable to conversations about school safety and staff wellbeing and collaboration beyond the United States. Only 40 states were represented in our qualitative sample. Furthermore, qualitative respondents represented only about half of the overall study sample (55.4%). Self-selection bias is possible and warrants consideration. Individuals more concerned about issues discussed in this article may have been more likely to respond than those who were generally satisfied with their school’s approach to safety and violence reduction. Our respondents’ demographics reflect what is generally known about the race, ethnicity, and gender of school social workers across the United States, with most being White women (Kelly et al., 2015). The current study’s sample precluded us from disaggregating participants by location or subgroups such as gender, race and ethnicity, career stage, and other characteristics.

### 4.7. Future Directions and Conclusions

These school social workers provided a unique perspective on the safety issues facing schools in the wake of the COVID-19 pandemic. Their responses highlight the complex needs of students and the comprehensive solutions required to address these needs through an ecological approach inclusive of families, schools, and the broader community. School social workers made several recommendations, including the importance of building trusting relationships in the school, emphasizing the role of relationships among students and between students and teachers or other adults. They also called for the intentional building of relationships between schools and community organizations to provide needed services for students and families and build trust between schools and families. Finally, school social workers drew attention to the need for system-level reforms: increased funding and staffing across various student support roles, increased general and specific training related to student well-being, and consistent implementation of discipline and safety policies bolstered by strong communication and collaboration among school staff members. The findings offer implications for practitioners and researchers alike, encouraging us to examine micro, meso, and macro solutions simultaneously to support positive student experiences in safe school communities.

## Figures and Tables

**Table 1:** Participant characteristics (N = 271).

Characteristic	*n*	%
Gender		
Female	241	88.93
Male	26	9.59
Nonbinary or other	4	1.48
Race		
African American	29	10.70
Asian	2	0.74
Hispanic	13	4.80
Multiracial	15	5.53
White	212	78.23
Urbanicity		
Suburban	108	39.86
Urban	106	39.11
Rural	57	21.03
Experience		
0–1 years	24	8.86
2–5 years	68	25.09
6–10 years	46	16.97
11–15 years	42	15.50
16–20 years	30	11.07
21–25 years	21	7.75
26–30 years	13	4.80
31+ years	8	2.95
No response	19	7.01
Grades served		
Elementary (pre-K, kindergarten, or Grades 1 through 4, 5, or 6)	79	29.15
Elementary (pre-K, kindergarten, or Grades 1 through 8 or 9)	12	4.42
Middle or junior high (Grades 5, 6, or 7 through 8 or 9)	47	17.34
High (Grades 9 or 10 through 12)	64	23.62
All (pre-K, kindergarten, or Grades 1–12)	34	12.55
No response	35	12.92

**Table 2:** Codebook example.

Category	Code	Definition	Subcode
What are the biggest safety issues facing educators and staff in your school?			
Issues related to external factors (community, outside of school)	Parents and families	Relationships between schools and families, and student life at home. Home is in the confines of family relationships.	
	Political and societal	Issues beyond the local community that affect safety, including political disagreements, human smuggling, etc.	
	Community	Relationships between schools and local community environments. Not related to family or household.	
	Weapon-related violence	Risk of weapon-related violence, including a mass shooting.	
Issues related to school policy, resources, and training	Lack of staff preparation to respond to student behaviors	Issues with training, policies, or resources needed in the school, e.g., training in de-escalation, better handling of discipline, etc.	
	Building security	Poor safety in school buildings, campuses, and door locks as school safety concerns, including lax behaviors toward security.	Physical plant (school structure)
			Problems with human behaviors related to building security, e.g., students leaving doors open or lax security.
			Not following protocols or plans (e.g., safety plans that are not used)
What policies, procedures, resources, or interventions are needed to better prevent or address violence in your school?			
School site policy and practice	Building school community and climate	Need for improved school environment and relationships between and within groups of students and staff members.	Climate and community
			Staff collaboration, e.g., use of staff members or how they engage with each other
			School–community connections (e.g., school–parent and school–family connections and communication)
			Administration–staff communication and support; specifically mentions administration, district, or leadership

**Table 3:** Findings—Themes and Subthemes.

Theme	Subtheme: Concern	Subtheme: Recommendation
Concerns with school safety require strengthening of school-community connections	Safety issues seen as external to the school	Need for stronger school-community connections to enhance safety
		Need for more positive school-family connections
Challenging student behavior requires interventions focused on building strong school community and climate	Students present with complex behavioral challenges	Complex behavioral challenges require ecological approaches
		A need to develop stronger school-based relationships
Concerns about student discipline, disruption, and defiance can be addressed with consistency, accountability, and improved systems of communication	Uneven distribution of consequences	Need for stronger and more transparent systems to navigate discipline
	Lack of communication and leadership around incidents	Need to develop stronger staff communication and support
Concerns about challenging student behaviors can be addressed with increased staffing and training	Staff feel overburdened in their roles and schools are understaffed	Hiring additional staff in a variety of roles
	Lack of training with advanced disciplinary approaches	The need for a variety of specific trainings to support staff effectiveness
		Bolstering special education staffing and support

## Data Availability

The data are not currently available for public access due to the size of the datasets, which are currently undergoing ongoing data cleaning, organizing, scale refinement, and scale validation. The study materials are available upon request.
